# Rocky Intertidal Zonation Pattern in Antofagasta, Chile: Invasive Species and Shellfish Gathering

**DOI:** 10.1371/journal.pone.0110301

**Published:** 2014-10-22

**Authors:** Juan Carlos Castilla, Patricio H. Manríquez, Alejandro Delgado, Verónica Ortiz, María Elisa Jara, Manuel Varas

**Affiliations:** 1 Centro de Conservación Marina, Estación Costera de Investigaciones Marinas, Las Cruces, Facultad de Ciencias Biológicas, Pontificia Universidad Católica de Chile, Santiago, Chile; 2 Laboratorio de Ecología y Conducta de la Ontogenia Temprana (LECOT), Centro de Estudios Avanzados en Zonas Áridas (CEAZA), Coquimbo, Chile; The Evergreen State College, United States of America

## Abstract

**Background:**

Biological invasions affecting rocky intertidal zonation patterns, yield information on species interactions. In the Bay of Antofagasta, northern Chile, the non-indigenous tunicate *Pyura praeputialis*, originally from Australia, has invaded (in the past century or so) and monopolized a major portion of the mid-intertidal rocky shore, displacing upshore the native mussel *Perumytilus purpuratus*. In Antofagasta the tunicate is subjected to intensive exploitation. Monitoring protocols show that in the past 10 years Antofagasta's tunicate population has experienced a drastic decline, affecting the intertidal zonation pattern.

**Methodology/Principal Findings:**

A 12.5 km of coastline, on the southern eastern shore of the Bay of Antofagasta, was studied. Eight sites were systematically (1993–1994) or sporadically (2003–2014) monitored for the seaward-shoreward expansion or reduction of the tunicate *Pyura praeputialis,* and native mussel and barnacle bands. A notable reduction in the mid-intertidal band of *P. praeputialis* and a seaward expansion of the mussel, *Perumytilus purpuratus*, and barnacle bands was observed. We suggest that the major cause for the decline in the tunicate is due to its intensive exploitation by rocky shore *Pyura*-gathers. The rate of extraction of tunicates by professional *Pyura*-gathers ranged between 256–740 tunicates hour^−1^. Between 2009–2014 the density of professional *Pyura*-gather ranged between 0.5–4.5 km^−1^ per low tide. Hence, 10 professional *Pyura*-gathers working 1 h for 10 low tides per month, during 6 months, will remove between 307–888 m^2^ of tunicates. A drastic decline in tunicate recruitment was observed and several *P. praeputialis* ecosystems services have been lost.

**Conclusion and Significance:**

In Antofagasta, the continuous and intensive intertidal gathering of the invasive tunicate *Pyura praeputialis,* has caused a drastic reduction of its population modifying the zonation pattern. Thereby, native mussel *Perumytilus purpuratus* has regained its ecological center in the intertidal zone. We recorded a *Pyura* recruitment failure and loss of ecosystem services.

## Introduction

One of the main goals of ecology is to understand the mechanisms and processes responsible for the abundance and distribution patterns of species in their environment, which includes humans. Rocky intertidal food-gathering by artisan or subsistence fishers affecting populations of carnivore keystone species, herbivores and primary producers, leading to the modification of population, community, trophic web and alternative ecological states, have been reported for central and southern Chile [1]–[7], South Africa [8]–[10] and Australia [11]. In these reports the ecological role played by humans, as resource extractors, has been characterized as extremely efficient and corresponding to a super-keystone species [12]. The study of non-indigenous invasive highly competitive species, in new environments, particularly if the invasive species is consumed, offers a unique opportunity for analyzing key ecological processes. In natural systems, rigorous ecologically designed experiments are not always possible due to the spatial scales involved, prohibitive logistics or ethical constraints. Instead, long-term monitoring and large-scale empirical observations can be used to determine the possible mechanistic (cause-effect) interactions among such impacts along with complexes of species.

Rocky intertidal non-indigenous invasive highly competitive sedentary species cause profound effects on shore biota, altering ecological balances [13]–[21]. Biological invasions can be seen as press bio-perturbations [22], resulting in a new ecological equilibrium. One of the best-documented examples is the rocky intertidal invasion of the mussel *Mytilus galloprovincialis* in South Africa [23]–[26]. Following the invasion, around 1979, the species rapidly dominated thousands of kilometers of exposed rocky shores along the west coast of South Africa, resulting in a modification of the intertidal zonation pattern, local biodiversity and causing a marked upshore displacement of the ecological center of gravity of the indigenous mussels *Choromytilus meridionalis* and *Aulacomya ater* [17].

Similarly, in the Bay of Antofagasta, Chile, it has been proposed that the intertidal and shallow subtidal barrel-shaped tunicate *Pyura praeputialis*, which has invaded and monopolized a major portion of the intertidal rocky shore, has changed the original intertidal zonation pattern [18]. *P. praeputialis*, originally from Australia, exhibits a conspicuous disjointed geographical distribution [27], in Chile it is known as “piure” and in Australia as “convejoy” [28], [29]. It has been argued that upon its arrival, probably more than 100 years ago [29], [30], the tunicate took over a large portion of the mid-intertidal zone (several meters of flat rocky platforms) and restricted the indigenous inferior competitor mussel *Perumytilus purpuratus* to the mid-upper intertidal fringe [18], [30]. This zonation pattern in the bay greatly contrasts with that observed in mid-intertidal fringes on other parts of the Chilean coast, where in the absence of the tunicate*s,* the mussel dominates the mid-intertidal primary substratum [1], [31], [32]. Environmental and ethical constraints [33], [34] have prevented us from undertaking large-scale removals (dozens or hundreds of meters) of tunicate or mussel inside the bay to test at a large-scale the inter-specific competitive hypothesis. Instead, we carried out small-scale manipulative and transplant field experiments, in caging units of less than 400 cm^2^, to test the competition strength and survival between *P. praeputialis* and *P. purpuratus* [18], [30]. The results show that in the mid-low intertidal fringe of the bay, where the tunicate fully dominates, it exhibits greater competitive strength than the native mussel. While further upshore, where the mussel dominates (mid-upper intertidal fringe), the survival of the tunicate is significantly reduced by the presence of the mussel.

Can the results of these small-scale experiments explain the overall intertidal zonation pattern inside the Bay of Antofagasta? In this paper we address this question, focusing on an area of 12.5 km of coast. We have taken advantage of monitoring information (decadal data series) on the intertidal zonation pattern of the tunicate, the mussel and barnacles and have combined that information with studies on the impact of intertidal extraction on *P. praeputialis* by food-gathers. Over the last few decades, probably as result of continuous and accumulative intertidal *Pyura*-gathering, a very substantial reduction in their matrices has occurred. The hypothesis to be tested is that under this large-scale decline (absence) of the invasive superior competitive dominant tunicate, a reinstatement of the original intertidal zonation pattern (as hypothesized as existing prior to the arrival of the tunicate) will occur. Thereby, beds of the inferior competitive native mussel *P. purpuratus* or stands the barnacles *Jehlius cirratus, Chthamalus scabrosus*, will expand down-shore (seaward), into the mid-low fringe, monopolizing rocky zones previously dominated by the superiorly competitive tunicate. Moreover, since in *P. praeputialis* settlement takes place mainly on the border of contiguous conspecifics or on the surface of the siphonal zone [35], we test the hypothesis that the severe reduction of *P. praeputialis* populations will be followed by a reduction in its recruitment in the study area.

## Materials and Methods

### Ethics statement

This long term research consisted solely of natural observations in fully public coastal areas, and data were collected in a manner that could not cause personal identification or harm. All reported results were obtained with informed oral consent prior to conducting observations and intertidal food-gathers and occasional gathers were given the opportunity to ask questions about our research, mainly regarding descriptions of hypotheses, goals and/or purposes of the study. If consent was not obtained, the observation was not made. We certify that when monitoring started in 1993 the Chilean research/academic system did not have in place any Institutional Review Board to approve the oral consent method described above.

### The Bay of Antofagasta and study sites

**Figure 1:**
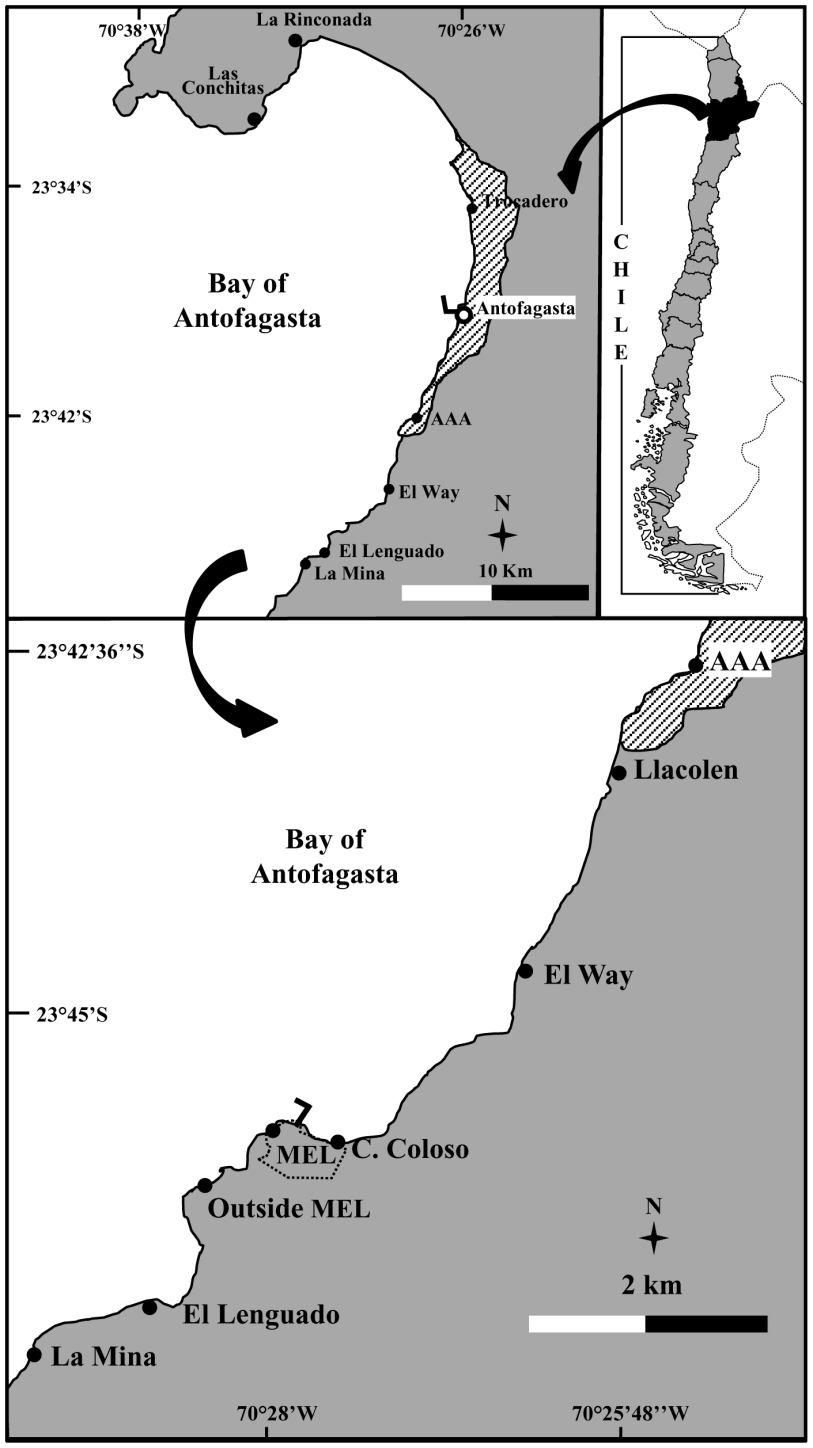
Map of the Bay of Antofagasta depicting the working area and main locations (see further details in reference 37).

The Bay of Antofagasta (23°36′S; 70°24′W) is a semi enclosed upwelling-shadow [36] embayment of about 70 km in length, with a 35 km wide southern facing mouth, between Punta Tetas in the north and Punta Coloso in the south (see oceanographic characteristics in [37]). The southeastern shore of the bay shows extended exposed intertidal flat rock platforms. The majority of the platforms in the area are reddish-brown continental sedimentary rocks, belonging to the Coloso formation, from the upper Jurassic or lower Cretaceous and to a lesser extent granitic rocks [38]. At Antofagasta there is a homogeneous semidiurnal tidal regime with a maximum extension of 1.68 to 1.78 m and all intertidal monitoring was done during diurnal low tides ranging between 0.20–0.40 m [39]. Our study area of 12.5 km is located along the southeastern shore of the bay: i) Asociación de Automovilistas de Antofagasta (AAA, 23°42′50″S; 70°25′43″W), ii) Llacolen (23°42′49″S; 70°26′00″W), iii) El Way (23°44″12″S, 70°26′ 28″W), iv) Caleta Coloso (23°45′23″S; 70°27′31″W), v) Minera Escondida Limitada at Punta Coloso (MEL, 23°45′17″S; 70°28′02″W), vi) Outside MEL (23°45′39″S; 70°28′21″W), vii) El Lenguado (23°46′12″S; 70°28′28″W), and viii) La Mina (23°46″42″S; 70°29′03″W) (Fig. 1). Sites were monitored monthly (1993–1994) and sporadically, 3–9 times per year (1997–2014) by a research team (same leader, JCC). Between 1989–1997 the rocky shore of MEL, approximately 2.5 km in length, was protected and fenced by the mining company and only occasional unauthorized visits by food-gathers were reported [40].

### Intertidal zonation in the bay and long-term monitoring

**Figure 2:**
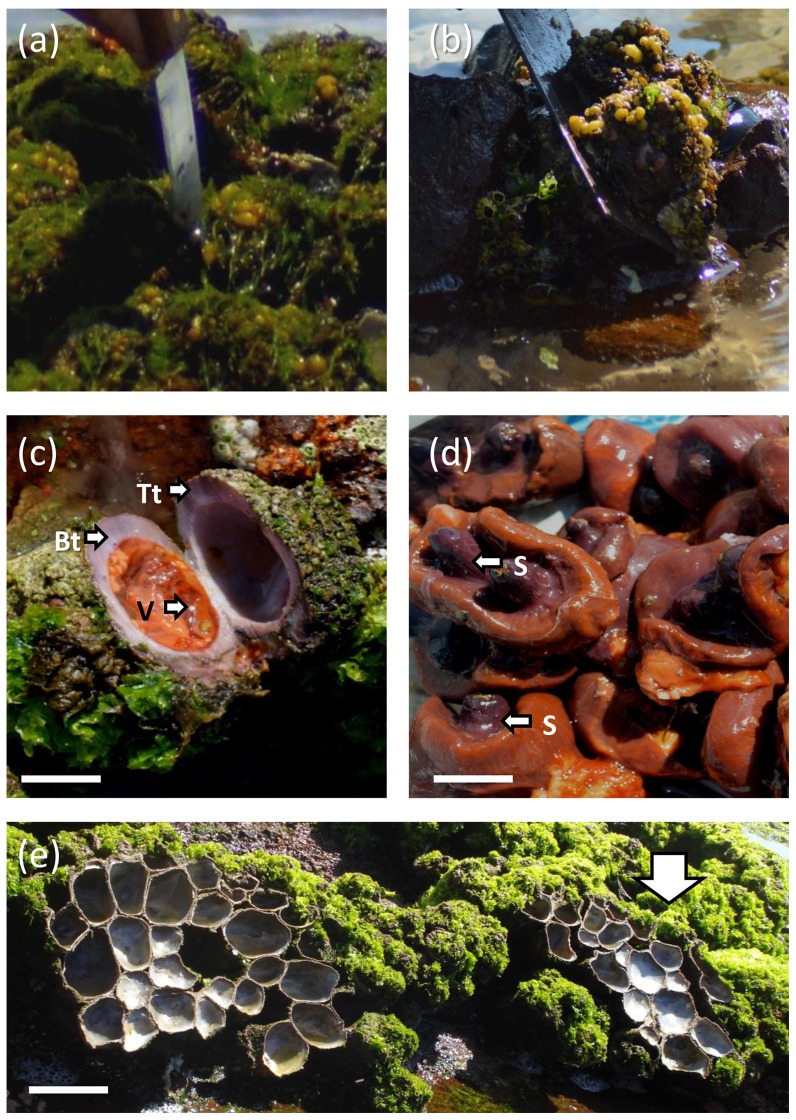
*Pyura praeputialis*. Photographic record of two extracting tools: (a) knife. (b) hand saw-blade. After the tough tunic is cut the top tunic is opened by hand to remove the edible parts or siphons (S). (c). The basal tunic (Bt) including the remains of the internal visceral tissues (V) and the top tunic (Tt) still connected with the basal tunic remains attached to the rocky substrate. (d) Only the upper parts of the soft tissues, mainly the siphons (S) are retrieved for consumption. (e) After a few hours local birds, fishes and wave action will remove the flesh and only the remains of contiguous tunics are observed. The green coloration (white arrow) on top of the matrices are the green algae *Ulva* spp. that grow on the siphonal zone of the tunicates. The scale bars  = 5 cm (c),  = 2 cm (d) and  = 10 cm (e).

The intertidal zonation pattern inside the Bay of Antofagasta, described for the first time in 1959 [41] is unique in Chile [42]. The upper intertidal band (covered only during high tide) consists of 90–95% bare rock and is characterized by abundant populations of the littorinids *Nodilittorina araucana* and *Nodilittorina peruviana*. Moving down-shore there is an intertidal mid-upper band dominated by dense tridimensional matrices of the mussel *P. purpuratus* [43], with a marked and constant landward limit (long-term observations using stainless hex head screw-bolts), likely set by exposure tolerance during low tides. Patches of the barnacles *Jehlius cirratus* and *Notochthamalus scabrosus* and algae such as *Ulva* spp., *Centroceras* spp., *Ceramium* spp. and *Colpomenia* spp. are also present. Lower down the mid-low band is dominated by dense matrices of the tunicate *Pyura praeputialis* that are frequently covered by an abundant growth of *Ulva* spp. (Fig. 2e). The height above sea level of the mid-intertidal *P. praeputialis* band in the different sampling sites ranged between 0.68 and 0.86 m above zero datum. The seaward limit is defined by the primary substratum cover of lithothamnioid/melobesioid algae (*Sinarthrophyton* spp., *Lithophyllum* spp.) and the barnacle *Australomegabalanus psittacus* [44]. The *P. praeputiali*s population structure, biomass and physical characteristics of matrices have been described [45], [46].

### Long-term changes in tunicate band width

To measure changes in the mid-low band width of *Pyura praeputialis* we monitored, between October 1993–October 1994, the distance between the landward and seaward edges of the band at 6 sites, from Llacolen to La Mina (Fig. 1; [47]. Sites were geo-referenced (hand-held Magellan Global Positioning System), photographed and mapped. Each site was 150–200 m long, separated by *ca.* 0.5–3 km and the monitored flat platforms had slopes ranging from 2° to 4°. Measurements were done monthly, during seven low tides, using 3–6 random replicates with a tape measure (0.5 cm accuracy). In January 2014 the same measurements were repeated. At this time, the *P. praeputialis* band was substantially reduced and the primary substrate was dominated by mussels (*P. purpuratus*) with the presence of barnacles (*Jehlius cirratus* and *Chthamamalus scabrosus*). Thus, this monitoring included measurements of the remaining tunicate band width plus data on the mussels and barnacles now installed covering the mid-low intertidal fringe. Additionally, in December 2003 and January 2014, we monitored (see above) changes in band width of the tunicate at three platforms with slopes <3° at AAA (Fig. 1). Platforms and intertidal fringes were marked with stainless hex head screw-bolts, photographed and mapped.

### Changes in mid-intertidal zonation patterns

From February 1999 to February 2014 (10 low tides) we monitored a single flat rock platform with a slope of <5° at El Lenguado (Fig. 1). This monitoring was done directly in the field or via photographs to measure the width of the mid-intertidal belt of *Perumytilus purpuratus* and that of *Pyura praeputials.* Additionally, from December 2003 to January-February 2014 (48 low tides), we monitored the same biotic fringe widths on three flat rock platforms at AAA and two flat rock platforms at El Way (slopes <3°) (Fig. 1). For photograph analysis we used Image Pro-Plus image analysis software.

### Recruitment of *P. praeputialis* monitoring

Censuses of new recruits of *P. praeputialis* were conducted during low tides in January and February of 2003 to 2008 and 2011 to 2014 on rocky intertidal platforms at El Way and AAA. *P. praeputialis* recruits were defined as individuals between 0.5–1.5 cm in maximum diameter. The count of recruits was done with the naked eye using a square quadrat of 0.04 m^2^ (0.20×0.20 m, n = 6 to 20) placed randomly on the matrices of adult individuals (>60% cover). In compact matrices (100% cover) the recruits were counted around the siphons; but when the cover was below 100% the counts also included recruits present on the lateral surfaces of adult individuals. A 1-way ANOVA followed by Tukey post hoc tests determined whether total number of recruits in each sampling locality varied among sampled years.

### Rocky intertidal food-gathering

Rocky shore food-gathering in Antofagasta is common and is carried out by professional and occasional (holidaymakers) gathers. Extractive activities take place during 7–10 low tides per month. From October 1993 to October 1994, we systematically monitored rocky shore food-gathers, along the 12.5 km study area [47]. At monthly intervals and over 7 days of low tides, the study area was monitored from a vehicle using binoculars every 0.5 km for 10–15 min, and the number of gathers recorded. Additionally, during 2009–2011 we occasionally (30 low tides) monitored the number of food-gathers present in the study area and during 2013–2014 we occasionally (20 low tides) monitored the number of food-gathers at Trocadero, north of the study area (Fig. 1). Monitoring was made particularly during January and February (austral summer), when the number of intertidal food-gathers in Antofagasta increases substantially due to the arrival of tourists [47]. The category of the extractors was recorded as: i) *Pyura praeputialis-*gather, targeting exclusively tunicates, ii) shellfish-gather, mainly targeting the muricid mollusk *Concholepas concholepas*, iii*) Octopus mimus-*gather, targeting exclusively octopuses, iv) skin diver operating from the rocky shore, v) anglers on the rocky shore. The activities were easily identified as the gathers use different extracting tools (i.e., knife, blade hand saw, curved iron rod or “chope”, or a long stick with a fake crab on the end to catch octopuses). For *P. praeputialis-*gathers we distinguish 3 major categories: i) professional *Pyura*-gathers, who use a sharp kitchen knife (Fig. 2a) to open tunicates one by one, including those extracted for consumption or for bait; ii) professional *Pyura*-gathers, who use a small blade hand-saw (Fig. 2b) to cut several *Pyura* at once (up to ¼ m^2^), including those extracting tunicates for consumption or for bait (Fig. 2d); iii) occasional *Pyura*-gathers, such as holidaymakers, that are less efficient extractors and can use knife, blade hand-saw or other tools. All *P. praeputialis* gathers make a transverse cut across the upper portion of the fibrous tunic of the tunicate to gain access to the edible siphon and upper portion of the soft body parts (Fig. 2c,d). The observations lasted for 20–160 minutes each and we recorded the time spent by the gathers on extractions and the number and size of tunicates extracted (caliper 1 mm accuracy). The tunicate extraction rate was standardized as the number of tunicates extracted per gather and per hour. The numbers of tunicates extracted per hour by professional and occasional gathers and the diameter of the extracted tunicates over the entire observation period and regardless of the sampling locality were compared by 1-way ANOVA followed by Tukey post hoc tests.

## Results

### Long term changes in tunicate band width

**Figure 3:**
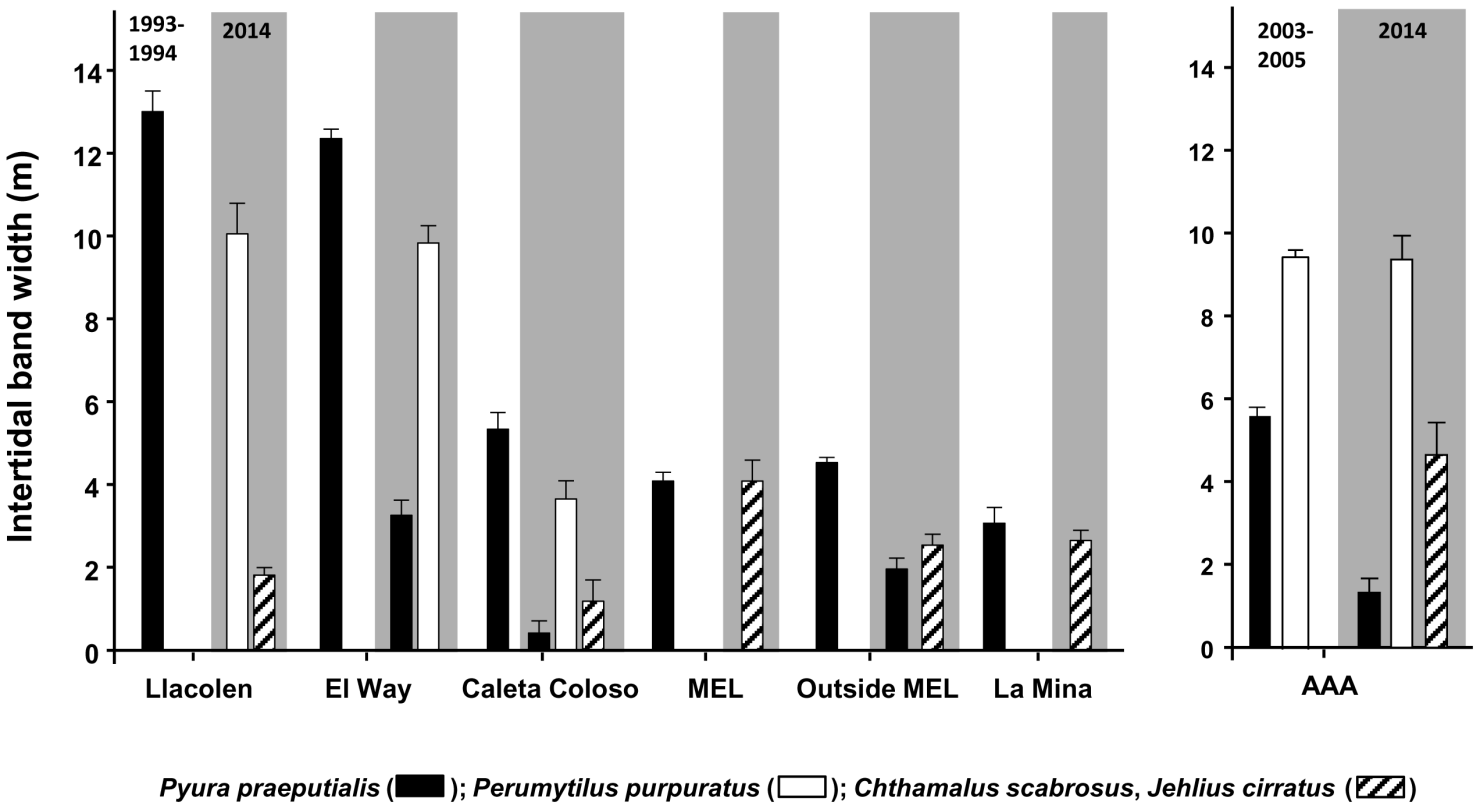
Mean intertidal band width (m; SE) of the tunicate *Pyura praeputialis* (black bars), the mussel *Perumytilus purpuratus* (white bars) and barnacles (*Chthamalus scabrosus*, *Jehlius cirratus*) (striped bars) at 7 sites in the bay of Antofagasta. For the first 6 sites the comparison is between 1993–1994 and 2014. For the seventh site the comparison is between 2003 and 2014 (b).

**Figure 4:**
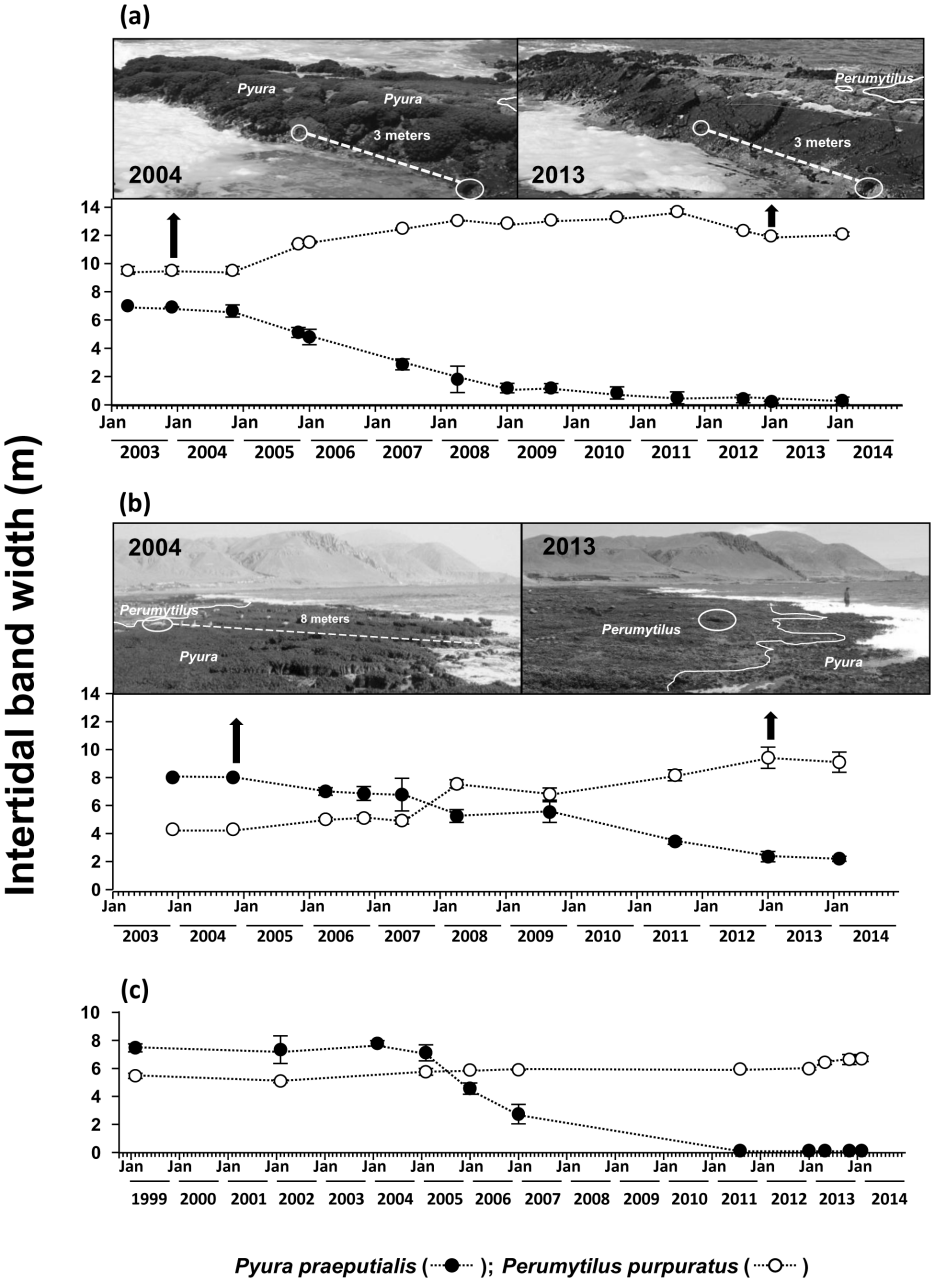
Temporal series of mean intertidal band width (m; SE) of the tunicate *Pyura praeputialis* (black circle) and the mussel *Perumytilus purpuratus* (white circle) in the bay of Antofagasta between April 2003 and February 2014 for a site at AAA (a) and a site at El Way (b) and between February 1999 and February 2014 for El Lenguado (c). For further details see text.

In the mid-intertidal zone, over time, a sustained and notable reduction of the intertidal band of *Pyura preaputialis* was observed (Fig. 3). Considering the 1993–1994 and 2014 sampling at Llacolen, MEL and La Mina, the tunicate intertidal band disappeared and the few tunicates that remained did not form continuous bands. At El Way, C. Coloso and Outside MEL the band width of *P. praeputialis* diminished between 60 and 90% (Fig. 3). In those sites, the characteristic mid-low intertidal band of tunicates has been totally or partially replaced by the mussel *P. purpuratus* and/or mixed stands of barnacles *J. cirratus* and *C. scabrosus* (Fig. 3). A similar situation was observed on three extra intertidal platforms at AAA and on two platforms at El Way. The sequential photographs (2003–2014) at these two sampling sites and at El Lenguado (1999–2014) show a dramatic modification of the zonation pattern in the mid-intertidal (Fig. 4a–c). At AAA the original 2003 tunicate band width of *ca*. 7 m (average) diminished, from 2003 onwards, reaching a width of 2 m in 2008 and had disappeared by 2014 (Fig. 4a). At El Way the original 2003 tunicate band width of *ca*. 8 m (average) diminished to around 2 m by 2014 (Fig. 4b). At El Lenguado, the original 1999 tunicate band width of *ca*. 8 m (average), started to diminish by 2006 and completely disappeared by 2014 (Fig. 4c). Conversely, at AAA and El Way the initial mussel band width increased to around 12 and 8 m respectively by 2014 (Fig. 4a–c). Mid-intertidal band monitoring at these three sites, shows rates of band width reduction or expansions that started around 2004–2006. This coincides with the observations at the intertidal sites monitored during 1993–1994 and during 2014 along the coast line analyzed (Fig. 3).

### Recruitment of Pyura praeputialis

**Figure 5:**
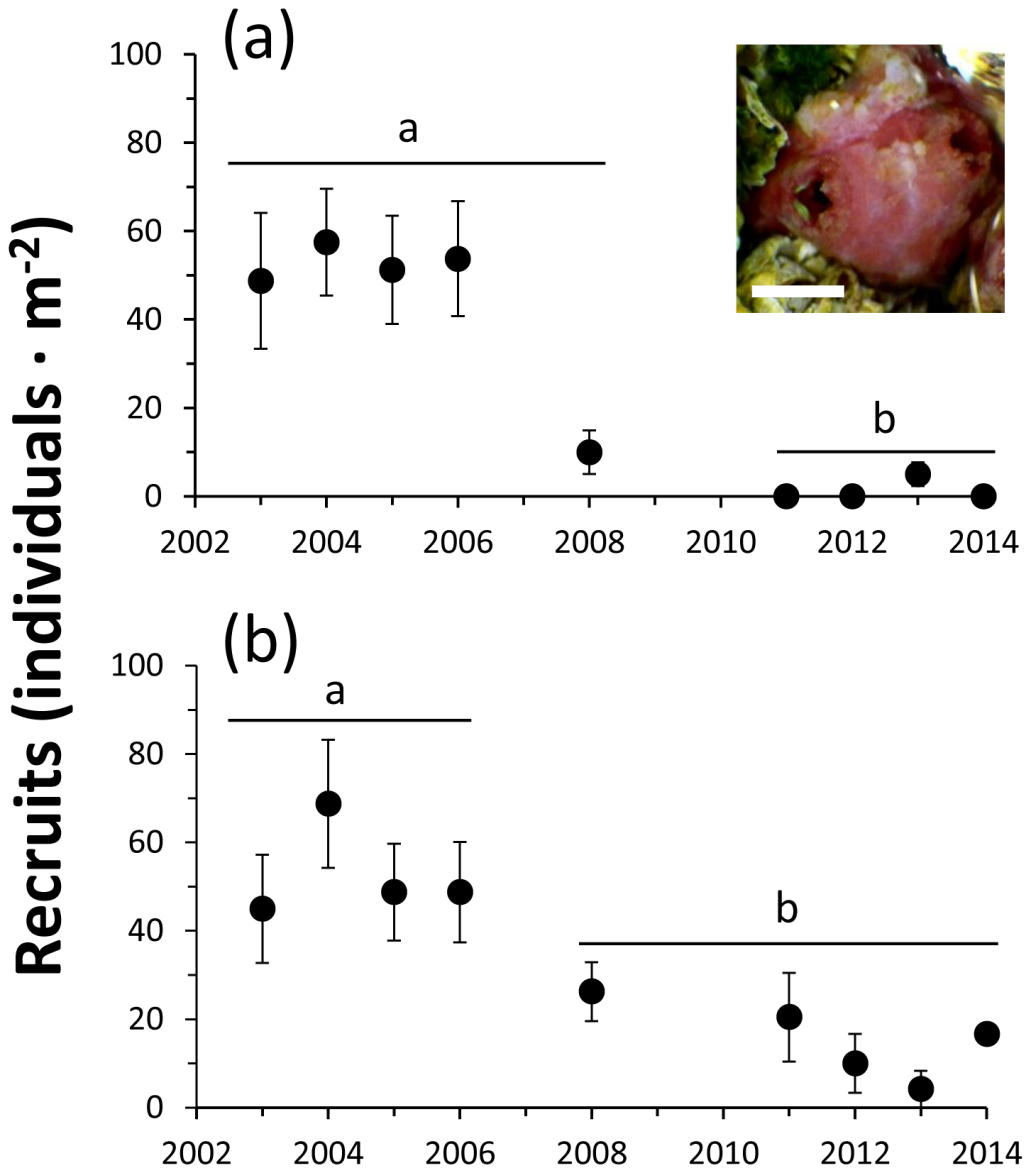
*Pyura praeputialis*. Temporal changes in the number (mean ±SE) of recruits (individuals m^−2^) recorded around the siphonal and basal area of conspecifics on rocky intertidal platforms at AAA (a) and El Way (b). Values with different letters depict mean values with significant differences. The inserted picture shows a small recruit in the siphonal area of an adult conspecific (scale bar  = 0.3 cm).

At El Way and AAA, during 2003–2014, two distinctive phases of recruitment abundances of *P. praeputialis* were distinguished (Fig. 5a–b). At El Way, during the first phase (2003–2008), the mean abundance of recruits was relatively constant, with 51.3 recruits m^−2^, this was followed (2011–2014) by a drastic drop, to practically no recruits (Fig. 5). At AAA, during 2003–2006, the mean abundance was *ca*. 48 recruits m^−2^, followed (2008–2014) by a drop to mean abundances of *ca*. 10 recruits m^−2^. The analyses detected that the mean recruit abundances were significantly higher during the first phase than during the second phase at both sampling sites (1-way ANOVA F_8,171_; 8.32; p<0.0001 and F_8,133_; 2.90; p<0.005 respectively; Fig. 5).

### Rocky shore shellfish and *Pyura praeputialis* extraction in Antofagasta

**Figure 6:**
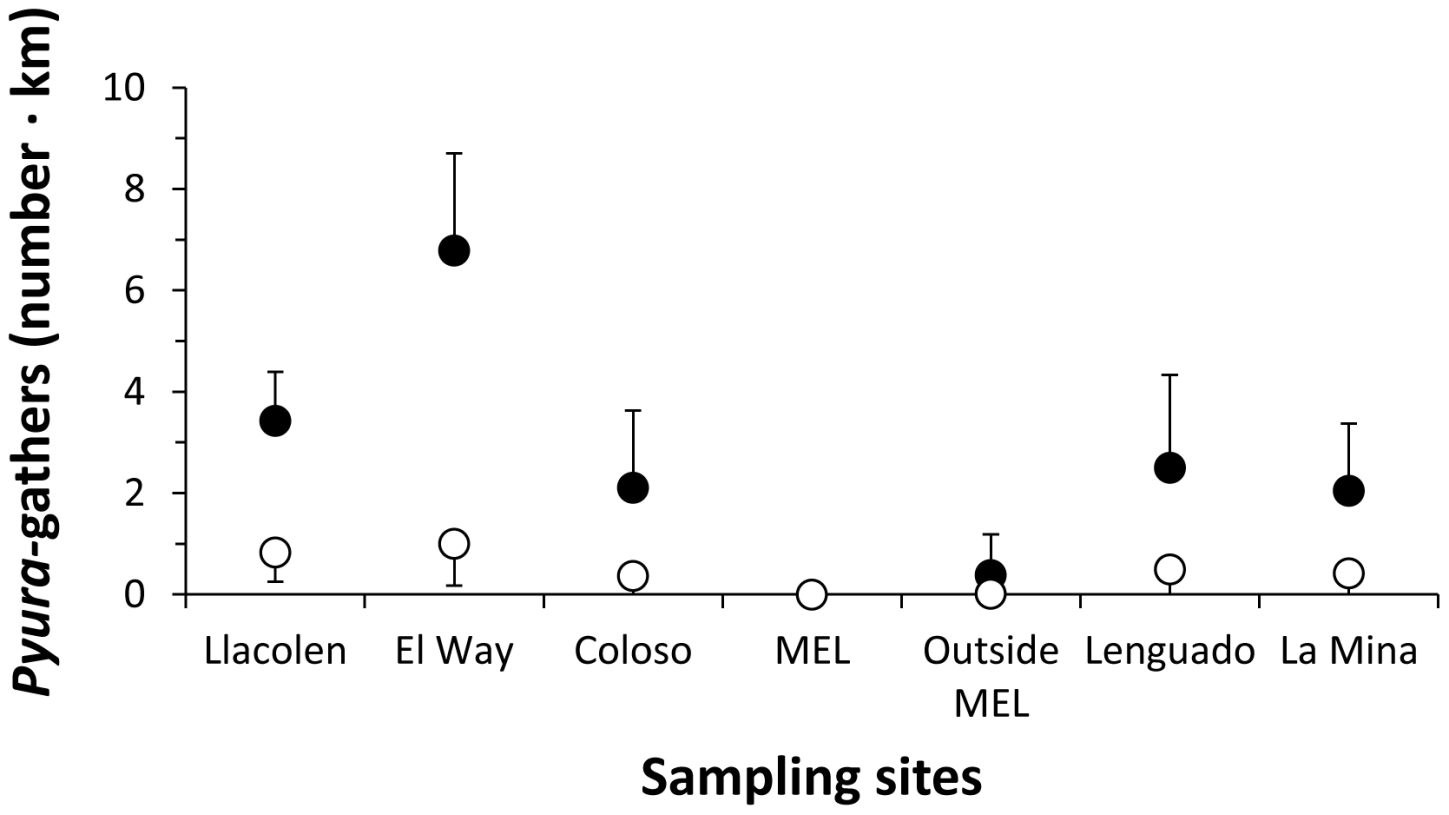
Mean number (±SD) of *Pyura*-gathers per km recorded over 13 months (1993–1994), in January and February (black circles) and the rest of the year (white circles) (taken and modified from 47).

The most frequent rocky intertidal extractors, during 1993–1994, were shellfish-gathers, with an overall mean per low tide of 1 individual km^−1^, followed by *P. praeputialis-*gathers with a mean of 0.86 km^−1^. They were followed by anglers at 0.56 km^−1^ and *Octopus*-gathers at 0.14 km^−1^ (-recalculated from [47] to fit the extension of our study site-). *P. praeputialis*-gathers exhibited a seasonal summer behavior concentrated during January and February. During those months, with the exception of MEL (about 2.5 km of fenced coastline, where no gatherers were recorded) the abundance of *Pyura*-gathers per low tide ranged from 6.8 km^−1^ in summer to 0.38 km^−1^ in winter (Fig. 6). At four intertidal sites (C. Coloso, El Way, AAA and Trocadero; Fig. 1), monitored during summer months (2009–2014), the mean abundance of professional *Pyura*-gathers ranged between 0.5–4.5 km^−1^ per low tide and the gathering excursion lasted from 6 to 120 min (Table 1). At these sites *Pyura*-gathers, using knives and blade hand-saws, extracted 256–740 tunicates hour^−1^ (Table 2). Regardless of the gathering tool and considering the entire study period the mean (±SD) extractions made by professional *Pyura*-gathers was 417.95 (±61.79) tunicates hour^-1^. This value was significantly higher than the 111.73 (±27.10) tunicates hour^−1^ extracted by occasional gathers over the same temporal period (1-way ANOVA F_1,46_ = 13,07; p<0.001). Moreover, the average size of the tunicates extracted by professional *Pyura*-gathers was significantly higher than for occasional gathers (1-way ANOVA; F_1,1646_ = 86,35; p<0.0001) (Table 2). Our conservative estimate of the removal of tunicates by professional *Pyura*-gathers (Tables 1, 2) indicates that 10 gathers working 1 h for 10 low tides per month, over 6 months, will remove between 153.360–444.000 tunicates; which would be equivalent to 307–888 m^2^ of compact tunicate matrices (Tables 1, 2; considering a mean density of 500 tunicates per m^2^ [46]. This estimate does not consider extractions made by holidaymakers or other less specialized food-gathers; information that is difficult to obtain.

**Table 1:** Standardized abundance of food-gathers and time spent on extraction activities in Antofagasta Bay (C. Coloso, El Way, AAA and Trocadero, Fig. 1) between 2009 and 2014.

Category	Abundance of gathers (number km^−1^ low tide)	Food gathering time (min)
*Pyura*-gathers		
Professional (knife)	0.5–2.5 (n = 10)	20–120 (n = 23)
Professional (saw)	0.5–2.5 (n = 4)	6–120 (n = 7)
Occasional	0.5–4.5 (n = 7)	30–80 (n = 14)
Shellfish-gathers		
Professional	1–4 (n = 3)	-
Occasional	3 (n = 1)	-

For further details see text.

**Table 2:** Average (±SD; N) descriptors of Pyura-gathers in Antofagasta Bay (C. Coloso, El Way, AAA, Trocadero, Fig. 1) divided into three temporal periods.

	Extraction (unit · h^−1^)	Mean diameter of the extracted *Pyura* (cm)
**(1993–1994)**		
*Pyura*-gathers		
Professional (knife & saw)	330.36 (8.78; 3)	8.57 (0.85; 752)
Occasional	220.76 (125.09; 5)	8.24 (1.03; 207)
**(2009–2011)**		
*Pyura*-gathers		
Professional (knife & saw)	474.22 (252.03; 5)	6.50 (1.89; 48)
Occasional	149.51 (49.29; 3)	5.81 (1.85; 31)
**(2013–2014)**		
*Pyura*-gathers		
Professional (saw)	740.51 (548.98; 7)	6.69 (2.20; 338)
Professional (knife)	256.31 (106.11; 13)	7.09 (1.67; 55)
Occasional	71.69 (50.57; 11)	6.16 (1.83; 196)

For further details see text.

## Discussion

Our monitoring along 12.5 km of shore on the southeastern border of Antofagasta Bay (Fig. 1), recording the width of the main intertidal bands, has shown that following the decline in the tunicate matrices, the intertidal zonation pattern inside the bay has drastically changed (Figs. 3, 4). Showing that changes in the zonation pattern reported in the present study had taken place across the entire sampling area. The native mussel and barnacles populations have relocated down-shore their ecological centers of abundance. The conspicuous mid-intertidal seascape dominated by the tunicate *Pyura praeputialis* has substantially decreased or disappeared and been replaced by mussel and/or barnacle bands. This shift is in line with predictions made by made by experimental small-scale caging and manipulative experiments which suggested that in the bay the invasive tunicate *P. praeputialis* outcompeted the native mussel in the mid-low fringe and thereby substantially modified the zonation pattern [18], [30]. We suggest that the major cause for the decline in the tunicate matrices in this area is the intensive and long-lasting exploitation, particularly by highly specialized and efficient intertidal rocky shore *Pyura*-gathers. This is the first large-scale demonstration that intertidal predatory human impacts can reverse the zonation pattern due to the invasion of a non-indigenous aggressive sedentary competitor. Of the various types of *Pyura* harvesters, professional gathers likely have the greatest impact. The use of the small blade hand saw is the extractive technique most detrimental for *P. praeputialis* matrices, because the removal is massive and patchy (up to 0.25 m^2^ per sawing; Fig. 2e). The extractive technique produces large openings in the matrices that weaken and expose matrices to further removal by mechanical forces associated with waves and storms, which are capable of removing large sections of the matrix [35], [48]. The knife extracting technique (Fig. 2a) is done individual by individual and therefore does less damage to the matrices. In Australia, [48] also concluded that substantial changes in density, for the same species, resulted from bait harvesting. Moreover, intertidal trampling may be another source of damage to the *Pyura* matrices. For instance, during the weekends of January and February 2014 in the study area we estimated 128–165 holidaymakers km^-1^ of coast, of which *ca*. 20–30% were camping and remained at the beaches beyond the weekends. During low tides (6 low tides, 20 h of observation over study area) holidaymakers were in the intertidal zone at numbers between 20–25 people km^−1^ of coast. At Antofagasta, there are social and economic incentives for the exploitation of tunicates. A professional *Pyura*-gather, working 7 days per month (total of 10–14 h) and selling *ca*. 150 kg of tunicates directly to restaurants can obtain *ca.* US$ 400–600. This is approximately the current official minimum monthly wage in Chile. In Antofagasta's fish markets raw *P. praeputialis* is sold at US$ 15–18 kg^−1^ and is considered a delicacy. Antofagasta is the fastest-growing city in Chile and tourism has flourished over the past 10 years, with increasing demands for marine products. Hence, over time, the tunicate intertidal matrix patchiness generated by intensive human gathering, waves, storms or predation [18], [35], [46], [49], may act additively or synergistically leading to severe matrix reductions. Nevertheless, other additive or synergistic causes for the reduction of this species, such as diseases [50], recruitment failure [48], natural predation [18], pollution or a reduction in the genetic variability in Antofagasta's isolated tunicate population cannot be ruled out.

Our results show a clear reduction in the abundance of recruits of *P. praeputialis*, which began around 2008, when the adult population was notably reduced (see Fig. 5). Given that settlement of *P. praeputialis* takes place mainly on the border of contiguous conspecifics or on the surface of the siphonal zone ([35], present study), it is likely that the severe reduction of intertidal populations of *P. praeputialis* has had negative consequences on the recruitment of the species. The drastic reduction in populations of *P. praeputialis* at Antofagasta has had also impacts on local biodiversity, since it has been shown that macro-invertebrate species richness is significantly higher in *P. praeputialis* matrices compared to *P. purpuratus* matrices [33]. Moreover, the existence of a dense *Pyura* band increases the abundance of intertidal populations of *Octopus mimus*, which inhabits the crevices between tunicates. *Octopus* gathering in the intertidal zone in the study area has almost completely ceased ([47], present study). A comprehensive analysis of the biological and ecological state of *P. praeputialis* populations inside the Bay of Antofagasta is urgently needed. This will help to determine whether or not the reported drastic reduction of intertidal populations of the tunicate in the analyzed area of the bay is generalized or not. Future research should aim to identify in the bay the existence of pristine *P. praeputialis* populations, which may serve to repopulate the rest of the bay, with the aim of conserving this unique non-indigenous species in Chile. It may sound controversial to advocate the conservation of a marine non-indigenous invasive species; nevertheless, in this case we think it makes sense since the invasive tunicate has increased the biodiversity of Antofagasta Bay [33] and provided numerous positive ecosystem services [20], [21], [47].
