# Correction: Rocky Intertidal Zonation Pattern in Antofagasta, Chile: Invasive Species and Shellfish Gathering

**DOI:** 10.1371/journal.pone.0113016

**Published:** 2014-11-03

**Authors:** The PLOS ONE Staff

Figure 4 is erroneously shown in black and white. The authors have provided a corrected, color version here.

**Figure 4:**
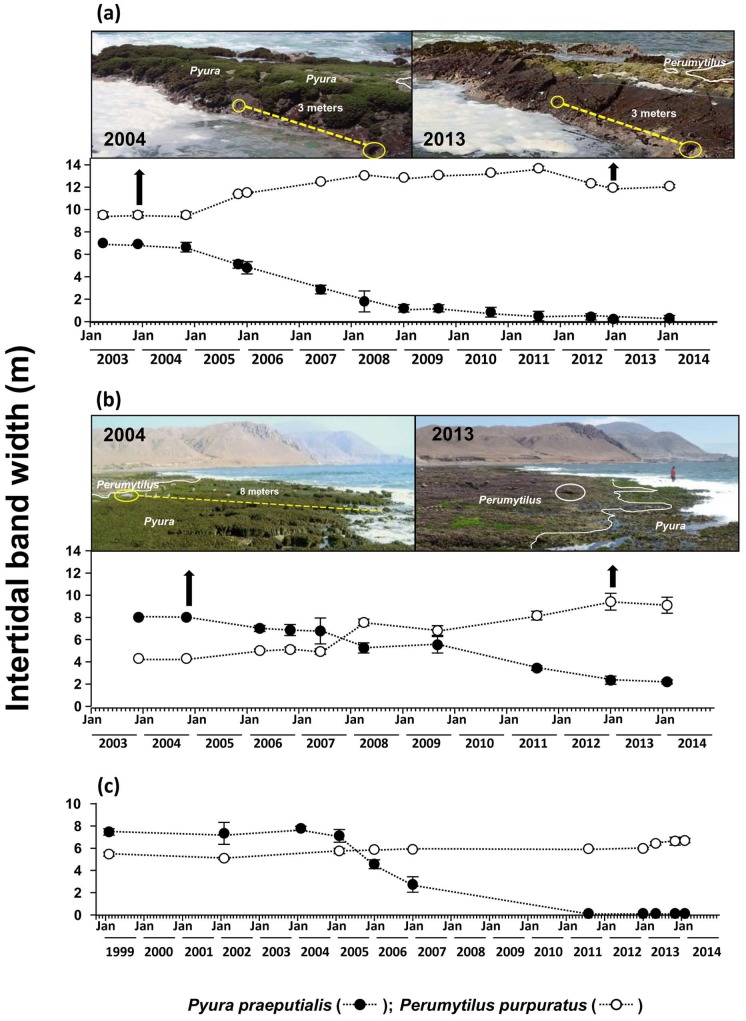
Temporal series of mean intertidal band width (m; SE) of the tunicate *Pyura praeputialis* (black circle) and the mussel *Perumytilus purpuratus* (white circle) in the bay of Antofagasta between April 2003 and February 2014 for a site at AAA (a) and a site at El Way (b) and between February 1999 and February 2014 for El Lenguado (c). For further details see text.

## References

[1] Castilla JC, Manríquez PH, Delgado A, Ortiz V, Jara ME, et al (2014) Rocky Intertidal Zonation Pattern in Antofagasta, Chile: Invasive Species and Shellfish Gathering. PLoS ONE 9(10): e110301 doi:10.1371/journal.pone.0110301 25338112 10.1371/journal.pone.0110301 PMC4206418

